# Identification of a sub-group of critically ill patients with high risk of intensive care unit-acquired infections and poor clinical course using a transcriptomic score

**DOI:** 10.1186/s13054-023-04436-3

**Published:** 2023-04-21

**Authors:** Maxime Bodinier, Guillaume Monneret, Marie Casimir, Aurore Fleurie, Filippo Conti, Fabienne Venet, Marie-Angélique Cazalis, Elisabeth Cerrato, Estelle Peronnet, Thomas Rimmelé, Anne-Claire Lukaszewicz, Karen Brengel-Pesce, Jean-François Llitjos

**Affiliations:** 1 Joint Research Unit HCL-bioMérieux, EA 7426 “Pathophysiology of Injury-Induced Immunosuppression” (Université Claude Bernard Lyon 1 – Hospices Civils de Lyon, bioMérieux), Lyon, France; 2 Open Innovation and Partnerships (OI&P), bioMérieux S.A., Marcy L’Etoile, France; 3 Immunology Laboratory, Edouard Herriot Hospital – Hospices Civils de Lyon, Lyon, France; 4 Centre International de Recherche en Infectiologie (CIRI), Inserm U1111, CNRS, UMR5308, Ecole Normale Supérieure de Lyon, Université Claude Bernard-Lyon 1, Lyon, France; 5 Anaesthesia and Critical Care Medicine Department, Hospices Civils de Lyon, Edouard Herriot Hospital, Lyon, France

**Keywords:** Sepsis, Transcriptomic, Intensive care unit, Acquired infections, Personalized medicine

## Abstract

**Background:**

The development of stratification tools based on the assessment of circulating mRNA of genes involved in the immune response is constrained by the heterogeneity of septic patients. The aim of this study is to develop a transcriptomic score based on a pragmatic combination of immune-related genes detected with a prototype multiplex PCR tool.

**Methods:**

As training cohort, we used the gene expression dataset obtained from 176 critically ill patients enrolled in the REALISM study (NCT02638779) with various etiologies and still hospitalized in intensive care unit (ICU) at day 5–7. Based on the performances of each gene taken independently to identify patients developing ICU-acquired infections (ICU-AI) after day 5–7, we built an unweighted score assuming the independence of each gene. We then determined the performances of this score to identify a subgroup of patients at high risk to develop ICU-AI, and both longer ICU length of stay and mortality of this high-risk group were assessed. Finally, we validated the effectiveness of this score in a retrospective cohort of 257 septic patients.

**Results:**

This transcriptomic score (TScore) enabled the identification of a high-risk group of patients (49%) with an increased rate of ICU-AI when compared to the low-risk group (49% vs. 4%, respectively), with longer ICU length of stay (13 days [95% CI 8–30] vs. 7 days [95% CI 6–9], *p* < 0.001) and higher ICU mortality (15% vs. 2%). High-risk patients exhibited biological features of immune suppression with low monocytic HLA-DR levels, higher immature neutrophils rates and higher IL10 concentrations. Using the TScore, we identified 160 high-risk patients (62%) in the validation cohort, with 30% of ICU-AI (vs. 18% in the low-risk group, *p* = 0.06), and significantly higher mortality and longer ICU length of stay.

**Conclusions:**

The transcriptomic score provides a useful and reliable companion diagnostic tool to further develop immune modulating drugs in sepsis in the context of personalized medicine.

**Supplementary Information:**

The online version contains supplementary material available at 10.1186/s13054-023-04436-3.

## Background

Sepsis is a complex life-threatening syndrome caused by a dysregulated host response to infection [1]. The primary inflammatory response is often followed by a complex and protracted immunosuppressive response affecting both the innate and adaptive components of immunity [2]. This post-septic immune suppression is associated with a higher risk of secondary infections, in the forefront of which are the intensive care unit (ICU)-acquired pneumonia [3]. However, a growing body of data suggest the existence of an important heterogeneity in the type and intensity of the immune response during post-septic immune suppression [4, 5]. Such heterogeneity is also retrieved at the patient level, sepsis from pulmonary origin being associated with a higher risk of subsequent ICU-acquired pneumonia when compared to sepsis from another origin [6]. Furthermore, the recent COVID-19 (Coronavirus disease 2019) pandemic has highlighted that the type of pathogen also influences the risk of ICU-acquired infection, the SARS-CoV-2 infected patients harboring a higher susceptibility to secondary pneumonia when compared to patients with flu or severe bacterial pneumonia [7, 8]. In this context, it is crucial to develop tools that allow for patient stratification according to their risk of ICU-acquired infections.

In this setting, transcriptomic approaches are promising for relevant informing stratification strategies in sepsis late phase. In a prospective cohort of septic patients, blood gene expression at the onset of ICU-acquired infections showed reduced expression of genes involved in gluconeogenesis and glycolysis [3]. In ICU patients with ventilator-associated pneumonia (VAP), authors reported transcriptomic depression of genes involved in the immunological synapse in the blood [9]. However, almost all studies investigate the transcriptomic response during the ICU-acquired infection rather than identify patients at risk to develop further infection. The recent development of multiplex molecular platforms such as the FilmArray System (bioMérieux) allows for rapid and reliable evaluation of the transcriptomic response of patients during nosocomial infections on the basis of the expressions of several genes involved in the pro- and anti-inflammatory response [10]. In this setting, we hypothesize that an analytical approach of gene expression, considering that each gene is mutually independent of the others and that they have a similar importance in the models, is relevant. We report here the diagnosis performance of a transcriptomic score performed between day 5 and day 7 after ICU admission that identify a subgroup of critically patients who are likely to exhibit poor clinical outcomes, including higher rates of ICU-acquired infection, longer ICU length of stay and higher mortality.

## Methods

### Patients and setting

In the training cohort, the patients and the data are abstracted from the previously published REALISM (REAnimation Low Immune Status Marker) cohort study [11]. Briefly, this monocenter observational cohort study included critically ill patients with sepsis, trauma and burn patients between December 2015 and June 2018. Inclusion criteria were: patients aged > 18 years, clinical diagnosis of sepsis as defined by the SEPSIS-3 consensus guidelines [1], severe trauma with injury severity score (ISS) > 15 or total burned surface area > 30%. Exclusion criteria were any of the following: presence of a preexistent condition or treatment that could influence patients’ immune status, pregnancy, institutionalized patients, inability to obtain informed consent. Whereas data and biological samples were collected at day 1 or 2 (D1-2), D3 or 4 (D3-4) and D5, D6 or D7 (D5-7), we focused on day 5–7. Longitudinal follow-up was performed for a period of 90 days. The IRB (Comité de Protection des Personnes Sud-Est II) approved the study (ref. 2015-42-2), and this study was registered at clinicaltrials.gov (NCT 02638779).

The validation cohort is derived from the MIPrea (Marqueurs Immunitaires Pronostiques en Réanimation) study [12] that enrolled patients aged > 18 years old with an expected length of stay > 2 days and SIRS criteria [13] between December 2009 and June 2011 in six French ICUs (approval CIC IRB #5044). This validation cohort consisted exclusively in patients with sepsis defined using the SEPSIS-2 definition [14]. In this study, we also analyzed biological data at day 5–7.

### Intended management

Septic patients were treated according to the guidelines of the Surviving Sepsis Campaign guidelines [15]. Patients received intravenous broad-spectrum antibiotics, depending on the presumed site of infection, previous antibiotic treatment and known colonization with antibiotic-resistant bacteria. Antimicrobial treatment was deescalated to narrower spectrum after identification of the responsible pathogen. Source control measures, such as surgery or removal of infected devices, were applied when necessary.

After initial stabilization using the “Parkland formula” for fluid resuscitation [16], burn patients were evaluated quickly to assess the need for debridement, escharotomy and fasciotomy in case of compartment syndrome. Opioids were used for pain control and sedation in addition to hypnotics. Patients underwent several surgical procedures for cleaning, non-viable tissue removal, skin graft and or amputation according to local aspect.

Initial management of severe trauma aimed to control the post-traumatic hemorrhage, including surgical procedures for damage-control and medical treatment of coagulopathy, and to stabilize hemodynamics with vasopressors if required. Patients were intubated and mechanically ventilated if alteration of consciousness, for preventing hypoxia. A restricted strategy of blood transfusion was applied and targeted hemoglobin of 70 to 90 g/L Pain management was based on opioids administration, combined with regional anesthesia if adapted, or associated with hypnotics in sedated patients. Patients could undergo several surgical procedures according to location of trauma [17].

### Definitions

Severity at admission was assessed by the Simplified Acute Physiology Score 2 and the Sequential Organ Failure Assessment (SOFA) scores in septic patients and using the ISS score in trauma patients [18–20]. ICU-acquired infections were defined as any new onset of probable or definite infection that developed after 48 h from ICU admission. Only the first episode of ICU-acquired infection was considered for analysis. ICU-acquired pneumonia was diagnosed according to the American Thoracic Society criteria [21]. Patients with clinically suspected ventilator-associated pneumonia were usually subjected to a tracheobronchial aspirate with semiquantitative cultures. Diagnosis of catheter-related bloodstream infection required the growth of the same pathogen from both peripheral blood and catheter tip cultures, or from blood cultures sampled from the catheter and from venous puncture with a differential time to positivity > 120 min. Urinary tract infections, mostly catheter related, were diagnosed upon the association of systemic manifestations of infection and positive urine bacterial culture at ≥ 10^5^ CFU/mL. An independent adjudication committee composed of three clinicians not involved in study patients’ recruitment or care reviewed the infections.

### Immunological measurements

The determination of the number of HLA-DR molecules per monocyte using the BD Quantibrite standardized method (HLA-DR: 340827; Quantibrite: 340495; Becton Dickenson, New Jersey, USA) was performed on fresh EDTA blood samples, within 3 h after collection. Plasma IL-10 level measurement was performed in ELISA using EDTA samples collected from patients.

### Immune multiplex molecular tool

The multiplex molecular tool was performed as previously reported [10]. Briefly, one PAXgene blood RNA tube (PreAnalytix, Hilden, Germany) was sampled at each time point, stabilized for at least 2 h at room temperature after collection and frozen at − 80 °C following manufacturer’s recommendations. mRNA expression was quantified using the FilmArray instrument (bioMérieux) for automatic mRNA reverse transcription, amplification and further quantitative nested PCR of twenty-six genes (*ADGRE3, ARL14EP, BPGM, C3AR1, CCNB1IP1, CD177, CD274, CD3D, CD74, CIITA, CTLA4, CX3CR1, GNLY, IFNG, IL10, IL1R2, IL1RN, IL7R, IP10/ CXCL10, MDC1, OAS2, S100A9, TAP2, TDRD9, TNF and ZAP70*). Of note, those twenty-six genes were selected on the basis of the literature [12, 22–27] and preliminary analysis from the REALISM study [11].

### Calculation of the transcriptomic score and statistical analysis

The normalized expression of individual transcripts of each gene evaluated by the multiplex molecular tool was first compared between healthy volunteers, patients with and without ICU-acquired infection. The receiver operator characteristics (ROC) curve for each gene to differentiate patient with and without ICU-acquired infection was then calculated. For genes with an area under the curve (AUC) > 0.70, we then calculated the optimal cut-point value as the value that maximizes the Youden’s index: sensitivity (%) + specificity (%) − 100. As sensitivity analysis, we additionally computed optimal cut-point using OOP (optimal operating point) method that is the value minimizing the distance from 100 of sensitivity and specificity: $\sqrt {\left( {100 - sensitivity} \right)^{2} + \left( {100 - specificity} \right)^{2} }$ [28]. The cut-point value of each gene is then used to define a threshold value above or below which (depending on its increase or decrease in patients with ICU-acquired infection) each gene is allocated or not a point. The transcriptomic score was therefore the sum of all the values for a given patient.

## Results

### Training cohort

In the REALISM cohort, 324 patients had a blood sampling at day 5–7. Among them, 176 did not presented with an ICU-acquired pneumonia before day 5–7 blood sampling and were still in the ICU at that time point. Most patients included in this training set were admitted for trauma (*n* = 72, 41%) and sepsis (*n* = 68, 39%). The overall ICU mortality was 8%.

**Fig. 1:**
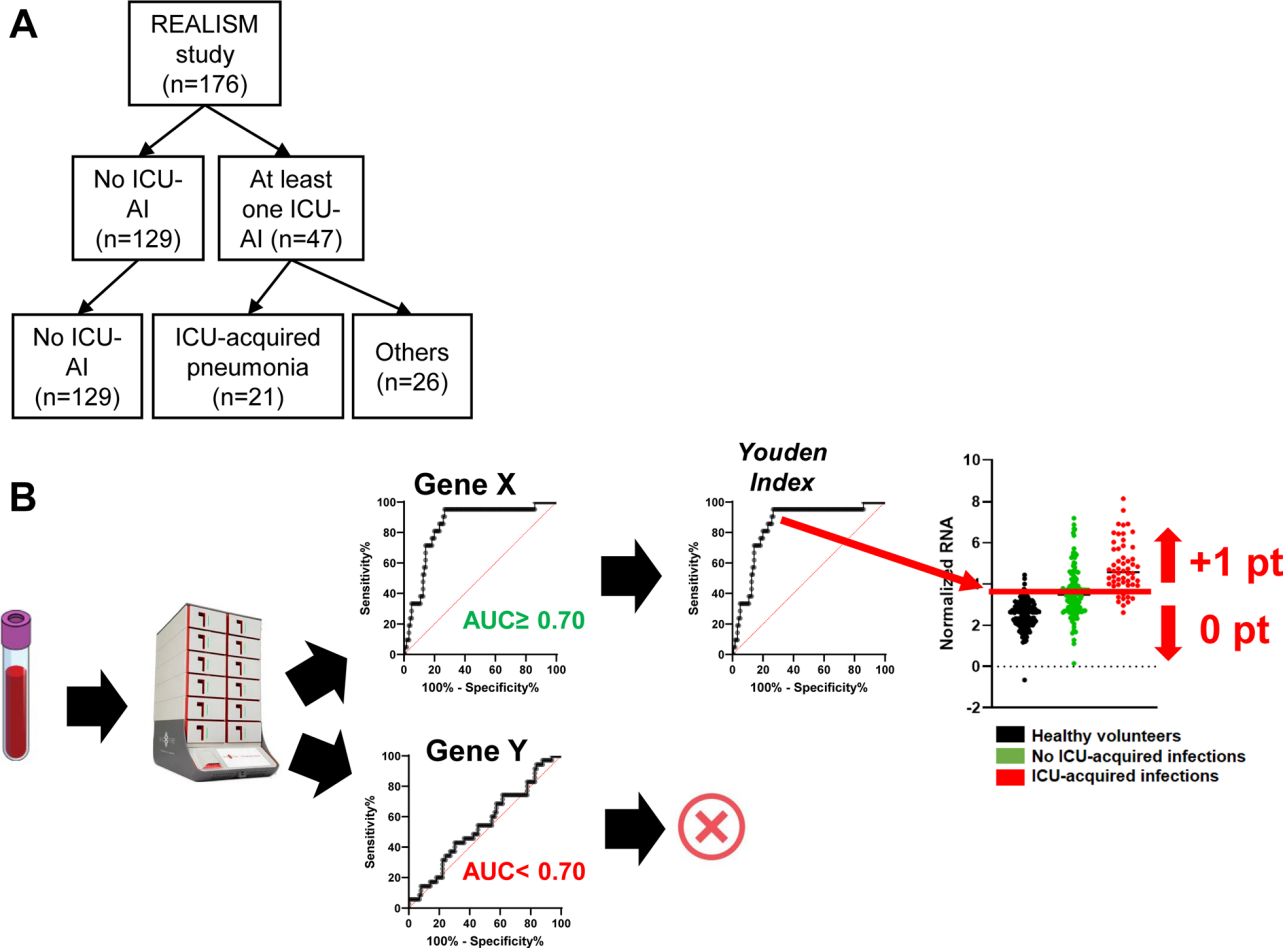
Characteristics and methods for the discovery cohort.** A** Flow-chart diagram of the discovery cohort, **B** Scheme depicting the method used to identify candidate genes

Overall, 47 patients presented at least one episode of ICU-acquired infection while 129 patients did not. Most ICU-acquired infections were ICU-acquired pneumonia (*n* = 21, 37%) (Fig. 1A). Patients who presented an ICU-acquired infection had more frequent diabetes mellitus (21% vs. 14%) and chronic pulmonary disease (19% vs. 9%) when compared to patients without ICU-acquired infections (Table 1). The median duration of mechanical ventilation was longer in patients with ICU-acquired infection when compared to patients without ICU-AI (15 days (95% IQR: 1–29) vs. 1 day (0–3), respectively).

**Table 1:** Clinical characteristics of the discovery cohort

	No ICU-AI (*n* = 129)	ICU-AI (*n* = 47)	*p*-value
*Baseline characteristics*			
Subgroup			
Burn	2 (2)	14 (30)	** < 0.001**
Sepsis/Septic shock	57 (44)	11 (23)	
Surgery	11 (9)	9 (19)	
Trauma	59 (46)	13 (28)	
Female gender	33 (26)	14 (30)	0.715
Age, years	61 (47–73)	59 (46–70)	0.446
Comorbidities			
Diabetes mellitus	19 (14)	10 (21)	0.420
Chronic renal failure	13 (9)	1 (2)	0.117
Chronic pulmonary disease	13 (9)	9 (19)	0.176
HIV infection	0 (0)	0 (0)	/
Solid tumors	22 (16)	9 (19)	0.921
Hematological malignancy	0 (0)	0 (0)	/
Body mass index, kg/m^2^	25 (22–28)	25 (22–29)	0.983
*Parameters at ICU admission*			
SAPS II score	35 (24–47)	38 (29–48)	0.401
SOFA score	6 (2–9)	7 (5–9)	0.134
Lactate, mmol/L	0.8 (0–2.6)	1.2 (0–2.6)	0.727
Mechanical ventilation	69 (53)	37 (79)	**0.004**
Norepinephrine or epinephrine use	88 (68)	40 (85)	**0.042**
*Outcomes*			
Duration of mechanical ventilation, days	1 (0–3)	15 (1–29)	** < 0.001**
Death in ICU	7 (5)	8 (17)	**0.028**
In hospital mortality	17 (13)	6 (13)	0.367

Bold values are statistically significant

**Fig. 2:**
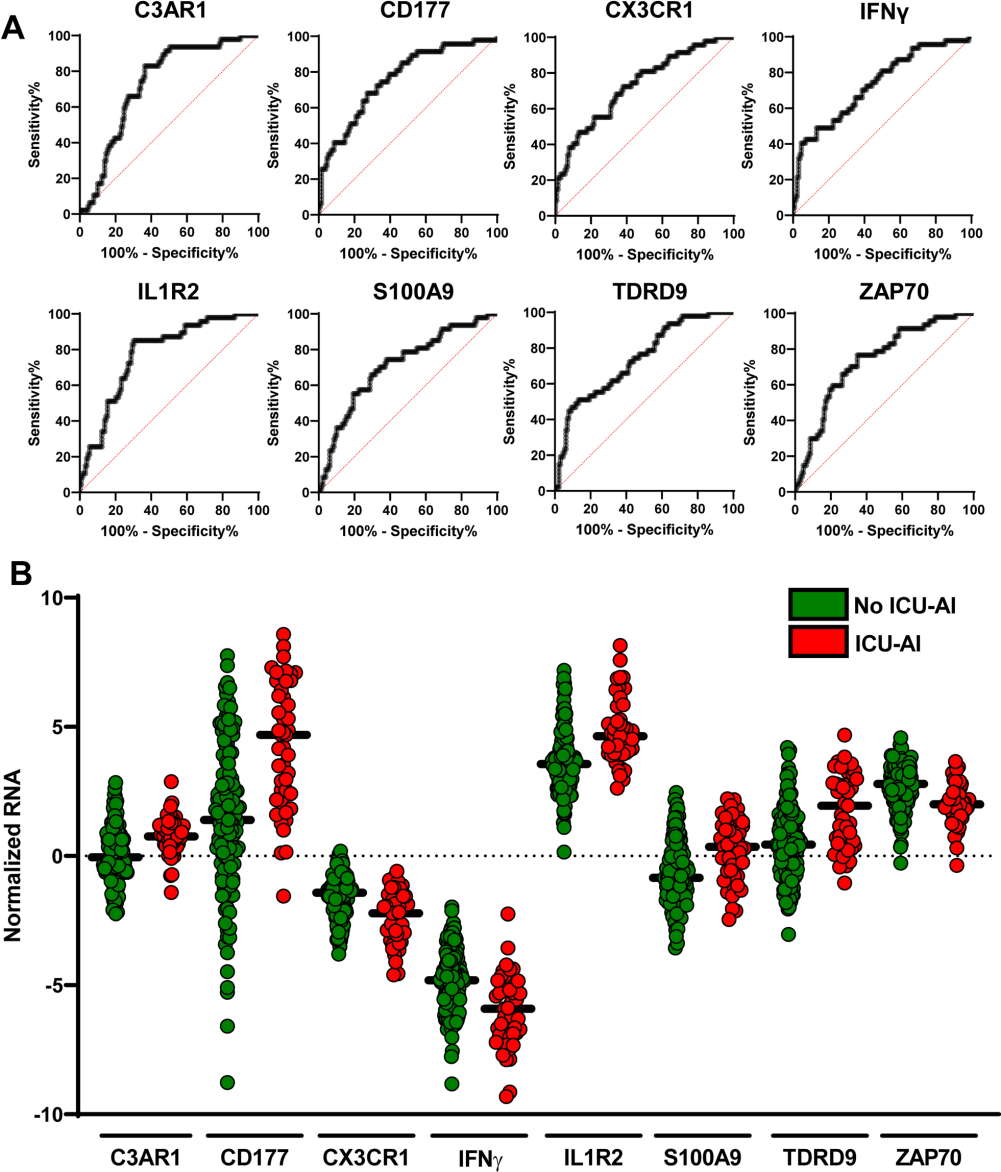
Gene’s identification process in the discovery cohort.** A** ROC (Receiver Operating Characteristics) curves with AUC (area under the curve) upper than 0.70 of several genes (C3AR1: Complement C3a Receptor 1, CD177: CD177 molecule, CX3CR1: C-X3-C motif chemokine receptor 1, IFNγ: Interferon gamma, IL1R2: Interleukin 1 receptor 2, S100A9: S100 calcium binding protein A9, TDRD9: Tudor domain containing 9, ZAP70: Zeta chain of T cell receptor-associated protein kinase 70), **B** Normalized RNA values for identified genes in patient with (red dots) or without (green) ICU-acquired infections (ICU-AI)

Using our approach (Fig. 1C), among the 26 genes included in the multiplex molecular tool, 8 transcripts had an AUC value upper than 0.70 to classify patients with or without ICU-acquired infection, namely *C3AR1*, *CD177*, *CX3CR1*, *IFNγ*, *IL1R2, S100A9, TDRD9* and *ZAP70* (Fig. 2A). Normalized mRNA transcript expression was increased in patients with ICU-acquired infections except for *CX3CR1, IFNγ* and *ZAP70* (Fig. 2B). The main diagnosis performances and cut-point value for each of the 8 transcripts are summarized in Table 2.

**Table 2:** Table summarizing diagnostic performances of identified genes in the discovery cohort

Genes	AUC	Cut-off value	Specificity	Sensitivity
C3AR1	0.73 (0.65–0.80)	> 0.360	0.63 (0.55–0.71)	0.83 (0.70–0.91)
CD177	0.76 (0.68–0.84)	> 2.867	0.73 (0.65–0.80)	0.68 (0.54–0.79)
CX3CR1	0.73 (0.64–0.81)	< − 1.679	0.62 (0.53–0.70)	0.72 (0.59–0.83)
IFNγ	0.73 (0.64–0.81)	< − 6.601	0.95 (0.90–0.98)	0.40 (0.27–0.55)
IL1R2	0.77 (0.70–0.84)	> 3.894	0.70 (0.61–0.77)	0.85 (0.72–0.93)
S100A9	0.71 (0.62–0.79)	> − 0.189	0.70 (0.62–0.77)	0.66 (0.52–0.78)
TDRD9	0.74 (0.65–0.82)	> 1.923	0.87 (0.80–0.92)	0.51 (0.37–0.64)
ZAP70	0.74 (0.65–0.81)	< 2.482	0.65 (0.57–0.73)	0.71 (0.50–0.86)

**Fig. 3:**
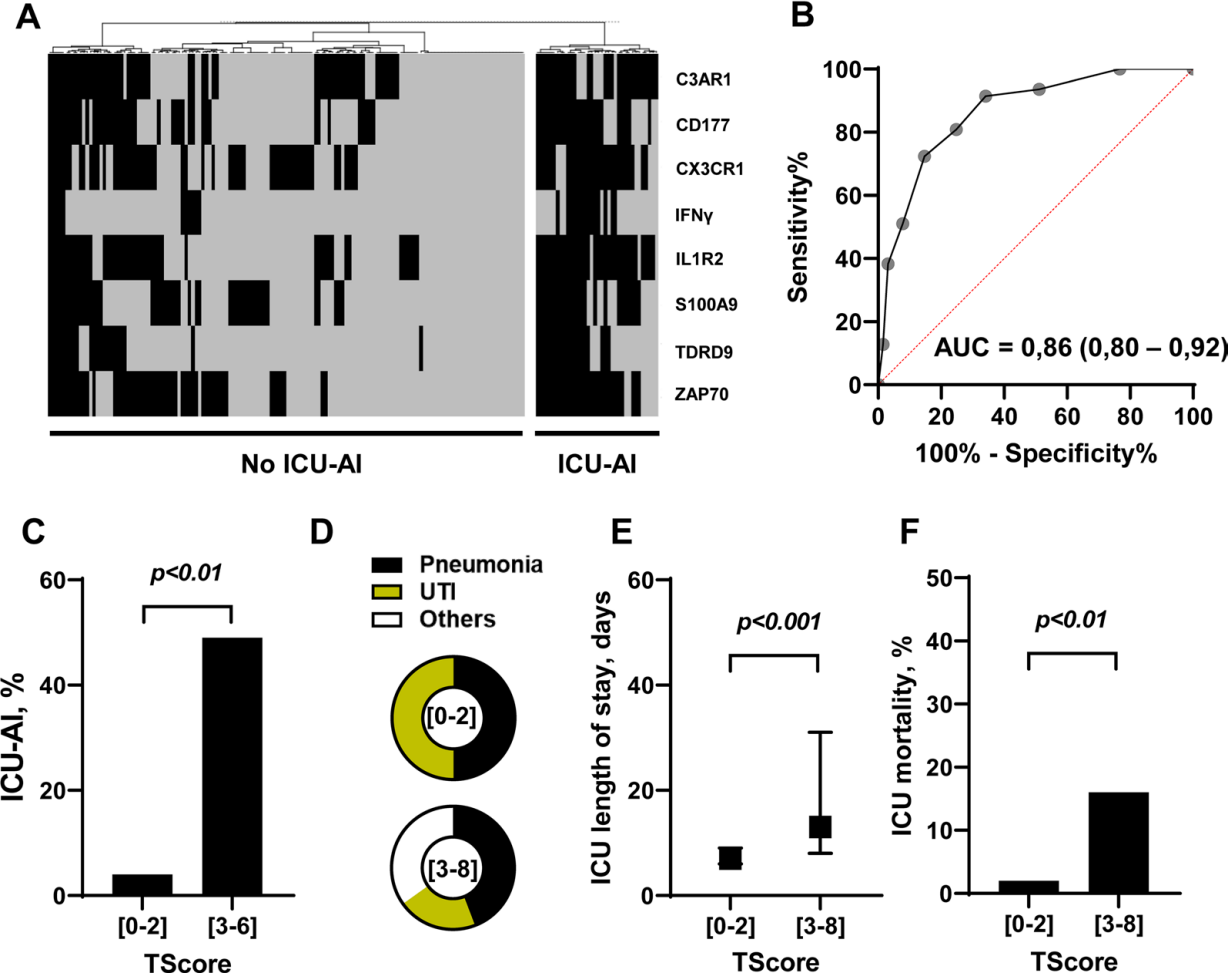
Clinical outcomes of high- and low-risk patients in the discovery cohort using the TScore. **A** Heatmap of unsupervised hierarchical clustering in patients with or without ICU-acquired infections using individual gene score, **B** ROC (Receiver Operating Characteristics) curve with AUC (area under the curve) value of the ability of the TScore obtained at day 5–7 to distinguish between patients that will develop or not at least an ICU-acquired infection during their ICU stay, **C** Intensive care unit acquired infection (ICU-AI) proportion in patients with a TScore between 0 and 2 (low risk) or upper or equal to 3 (high risk), **D** Distribution of the type of ICU-AI in patients with a TScore between 0 and 2 (low risk) or upper or equal to 3 (high risk) (UTI: urinary tract infections), **E** Median and interquartile duration of intensive care unit length of stay in patients with a TScore between 0 and 2 (low risk) or upper or equal to 3 (high risk), **F**: intensive care unit mortality rate in patients with a TScore between 0 and 2 (low risk) or upper or equal to 3 (high risk)

We built the transcriptomic score (TScore) using the threshold values obtained with the Youden technique on the ROCs for each transcript. For each gene analyzed individually, patients with a value above the threshold (for *C3AR1*, *CD177*, *IL1R2, S100A9* and *TDRD9*) or below threshold (for *CX3CR1, IFNγ* and *ZAP70*) were assigned a point. Conversely, genes that do not meet these criteria were assigned a value of 0. The TScore, which varies between 0 and 8, is the sum of the values for each of the 8 genes (Fig. 3A). The AUC value of this TScore performed at day 5–7 is 0.86 (0.80–0.92) in distinguishing patients with or without at least one episode of ICU-acquired infection during their ICU stay (Fig. 3B). As sensitivity analysis, the OOP approach retrieved similar results but with lower ROC AUC for TScore (Additional file 1: Table S1 and Fig. S1).

Using the Youden index, the optimal value of the score is 2. We therefore defined a high-risk population of patients with a TScore upper to 2 and a low-risk population of patients with a TScore lower or equal to 2.

When applied to the whole population of the training set, patients with a TScore between 3 and 8 (*n* = 87, 49%) presented a higher proportion of ICU-acquired infection when compared to patients with a TScore lower or equal to 3 (49% vs. 4%, respectively) (Fig. 3C). In these high-risk patients, ICU-acquired infections were more frequently pneumonia (*n* = 19, 49%) when compared to low-risk patients (*n* = 2, 11%) (Fig. 3D). Furthermore, they experienced a longer median ICU length of stay (13 days, 25th–75th IQR: 8–30 vs. 7 days, 25th-75th IQR: 6–9, *p* < 0.001) when compared to patients in the low-risk TScore group (Fig. 3E). The ICU mortality in the high-risk TScore group was higher than the mortality of the patients in the low-risk TScore group (15% vs. 2%, *p* < 0.01) (Fig. 3F). All parameters are summarized in Table 3.

**Table 3:** Table summarizing the clinical characteristics and outcomes of patients with a TScore between 0 and 2 (low risk) or upper or equal to 3 (high risk) (SOFA: Sequential organ failure assessment score)

	TScore [0–2] (*n* = 89)	TScore [3–8] (*n* = 87)	*p*-value
*Baseline characteristics*			
Subgroup			** < 0.001**
Burn	0 (0)	16 (18)	
Sepsis/Septic shock	29 (33)	39 (45)	
Surgery	5 (6)	15 (17)	
Trauma	55 (62)	17 (20)	
Female gender	20 (22)	27 (31)	0.266
Age, years	54 (43–66)	65 (49–76)	**0.004**
Body mass index, kg/m^2^	25 (23–27)	26 (23–30)	0.314
*Parameters at ICU admission*			
SAPS II score	34 (23–41)	43 (30–54)	** < 0.001**
SOFA score	5 (1–8)	8 (5–11)	** < 0.001**
Lactate, mmol/L	0 (0–2.1)	1.6 (0–3.2)	0.078
Mechanical ventilation	41 (46)	65 (75)	** < 0.001**
Norepinephrine or epinephrine use	54 (61)	74 (85)	** < 0.001**
*Outcomes*			
ICU length of stay, days	7 (6–9)	13 (8–30)	** < 0.001**
ICU-acquired infections	4 (4)	43 (49)	** < 0.001**
Pneumonia	2	19	
Urinary tract infection	2	9	
Others	0	15	
Death in the ICU	2 (2)	13 (15)	**0.006**
In hospital mortality	9 (10)	14 (16)	0.114

Bold values are statistically significant

In the training set, a Wilcoxon–Mann–Whitney test was performed to determine potential relevant clinical and biological variables of interest for predicting the occurrence of ICU-acquired infections. Of the variables analyzed, only SOFA score at day 6 and T score showed a statistically different distribution between ICU-acquired infections and no ICU-acquired infections. In a multivariate model, the TScore remained independently associated with ICU-acquired infections occurrence (Table 4).

**Table 4:** Univariate analysis of relevant clinical parameters between low-risk and high-risk patients in the discovery cohort followed by the multivariate analysis including parameters statistically significant in univariate analysis

Univariate analysis	*p*-value
Age, years	0.89
Charlson score	0.66
SAPSII score	0.46
SOFA at day 6	0.0079
TScore	3.8 × 10^–9^

Multivariate analysis	Estimate	*p*-value
SOFA at day 6	− 0.06	0.32
TScore	0.6	6.4 × 10^–9^

**Fig. 4:**
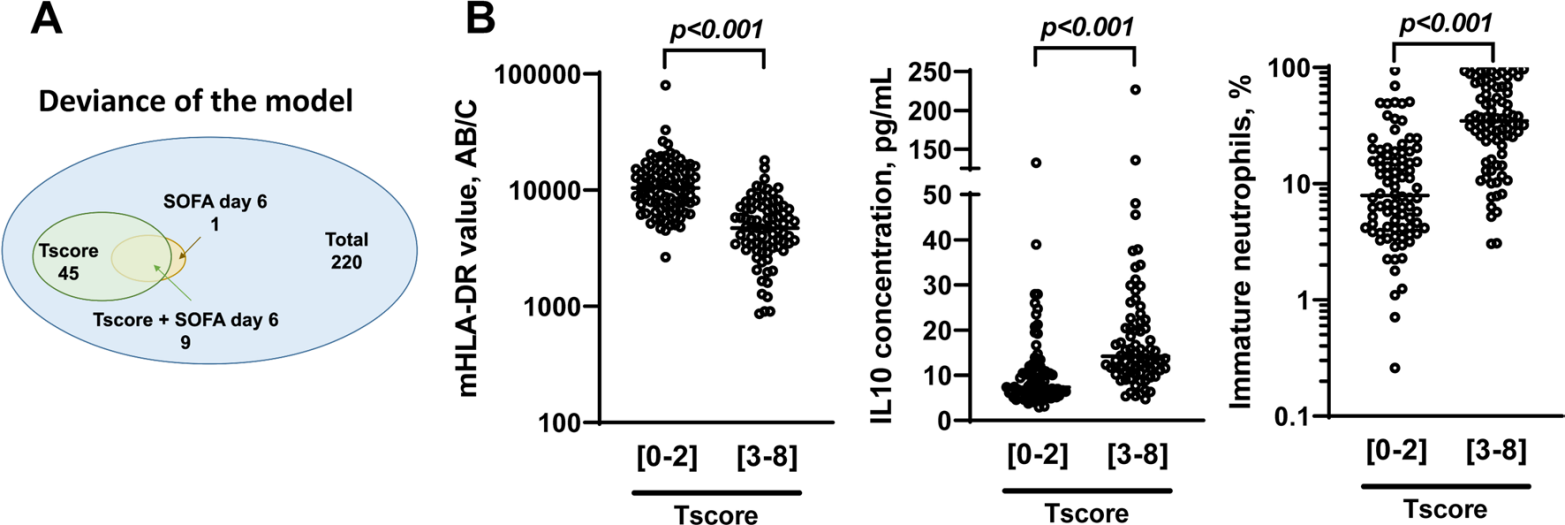
Statistical and immunological validity of the TScore in the discovery cohort. **A** picture representing the respective participation of each parameter in the deviance of the model and their overlap, **B** level of expression of several immunological parameters (monocytic HLA-DR, IL-10 and immature neutrophils proportion) measured in blood of patients with a TScore between 0 and 2 (low risk) or upper or equal to 3 (high risk) and **C** clinical parameters and outcomes of patients with a TScore between 0 and 2 (low risk) or upper or equal to 3 (high risk) sub-classified according to their subgroup

A type I ANOVA was applied, varying the order of introduction of the explanatory variables in the model, to determine the own contribution of the explanatory variables TScore and SOFA at day 6 to the minimization of deviance, as well as their joint contribution. The model thus created explains 25.4% of the total deviance, with 81% being explained by the TScore alone, 16.8% of the deviance explained by the TScore and the SOFA at day 6 together, and 1.7% by SOFA at day 6 alone. TScore therefore only slightly overlaps with information already explained by the main clinical variables in the REALISM database and the addition of such variables to TScore seems of little relevance in regard of the low proportion of deviance they explained in the model (Fig. 4A).

Finally, we investigated the expression level of several biomarkers previously reported to be associated with poor outcomes in ICU patients [29]. Interestingly, we found that the monocytic HLA-DR level of expression was decreased in high-risk patients. Similarly, the concentration of the anti-inflammatory cytokine IL10 and the immature neutrophils rate was increased in high-risk patients (Fig. 4B and Additional file 1: Table S2). Finally, when we apply our model to the training cohort according to the initial aggression (sepsis, burns, trauma and surgery), we find the same capabilities of the TScore to identify a group at high risk of complications (Table 5).

**Table 5:** Characteristics of patients according to their first hit location

	Burn		Sepsis			Surgery			Trauma		
	Low Risk (*n* = 0)	High Risk (*n* = 16)	Low Risk (*n* = 29)	High Risk (*n* = 39)	*p*	Low Risk (*n* = 5)	High Risk (*n* = 15)	*p*	Low Risk (*n* = 55)	High Risk (*n* = 17)	*p*
SAPS II at admission	/	37 [29–42]	40 [36–49]	53 [46–62]	** < 0.001**	26 [25–27]	25 [22–31]	0.521	26 [21–38]	35 [29–45]	**0.042**
SOFA at admission	/	6 [5–8]	8 [6–10]	10 [8–12]	**0.002**	3 [3, 4]	5 [2–6]	0.347	2 [0–7]	7 [3–11]	**0.008**
Lactate at admission	/	1.9 [0.0–3.6]	1.3 [0.0–2.1]	2.5 [0.0–3.6]	**0.040**	0.0 [0.0–0.0]	0.0 [0.0–0.0]	/	0.0 [0.0–2.1]	1.6 [0.0–2.8]	0.150
Mechanical ventilation	/	14 (87.5%)	20 (69.0%)	34 (87.2%)	0.125	0 (0.0%)	3 (20.0%)	0.539	21 (38.2%)	14 (82.4%)	**0.004**
Norepinephrine or epinephrine use	/	14 (87.5%)	27 (93.1%)	38 (97.4%)	0.571	2 (40.0%)	9 (60.0%)	0.617	25 (45.5%)	13 (76.5%)	**0.050**
ICU length of stay, days	/	53 [39–62]	7 [6–8]	13 [10–19]	** < 0.001**	7 [6, 7]	8 [6–9]	0.056	8 [6–9]	18 [12–33]	** < 0.001**
Mechanical ventilation duration, days	/	26 [16–44]	3 [0–5]	3 [1–8]	0.201	0 [0–0]	0 [0–0]	0.326	0 [0–1]	5 [1–23]	** < 0.001**
Death in the ICU	/	4 (25.0%)	2 (6.9%)	7 (17.9%)	0.282	/	/	/	0 (0.0%)	2 (11.8%)	0.053
In hospital mortality	/	3 18.8%)	8 (27.6%)	10 (25.6%)	1.000	/	/	1.000	1 (1.8%)	1 (5.9%)	0.419
ICU-acquired infections	/	14 (87.5%)	0 (0.0%)	11 (28.2%)	**0.002**	0 (0.0%)	9 (60.0%)	**0.038**	4 (7.3%)	9 (52.9%)	** < 0.001**

Bold values are statistically significant

*LOS* Length of stay, *MV* Mechanical ventilation

### Validation cohort

For the validation cohort, we assessed the diagnosis performance of the TScore in septic patients from the MIPrea cohort. The proportion of high-risk patients with a TScore between 3 and 8 was higher in the MIPrea cohort when compared to the REALISM cohort (62% vs. 49%, respectively). High-risk patients were most severe at ICU admission, with a higher SOFA score (10, 25th–75th IQR: 7–13 vs. 8, 25th–75th IQR: 5–11) and higher rate of mechanical ventilation (87% vs. 75%) (Table 6).

**Table 6:** Table summarizing the clinical characteristics and outcomes of patients with a TScore between 0 and 2 (low risk) or upper or equal to 3 (high risk)

	TScore [0–2] (*n* = 97)	TScore [3–8] (*n* = 160)	*p*-value
*Baseline characteristics*			
Female gender	34 (35)	63 (39)	0.575
Age, years	64 (54–75)	66 (57–77)	0.414
Comorbidities			
Diabetes mellitus	22 (22)	25 (15)	0.211
Chronic renal failure	10 (10)	11 (7)	0.460
Chronic pulmonary disease	32 (33)	44 (27)	0.427
HIV infection	1 (1)	1 (1)	1.000
Solid tumors	19 (20)	44 (28)	0.201
Hematological malignancy	5 (5)	1 (1)	**0.030**
*Parameters at ICU admission*			
SAPS II score	56 (40–69)	60 (48–70)	0.061
SOFA score	8 (6–10)	10 (8–13)	** < 0.001**
Lactate, mmol/L	1.8 (1.2–2.9)	2.4 (1.6–3.6)	** < 0.001**
Mechanical ventilation	75 (77)	140 (87)	**0.049**
Norepinephrine or epinephrine use	62 (64)	123 (77)	**0.036**
*Outcomes*			
ICU length of stay, days	9 (7–14)	12 (8–20)	**0.003**
Duration of mechanical ventilation, days	6 (3–14)	9 (5–16)	**0.011**
ICU-acquired infections	18 (18)	47 (30)	0.056
Pneumonia	10	32	0.134
Urinary tract infection	6	8	
Others	2	7	
Death in ICU	13 (13)	47 (29)	**0.006**

Bold values are statistically significant

*SOFA* Sequential organ failure assessment score

**Fig. 5:**
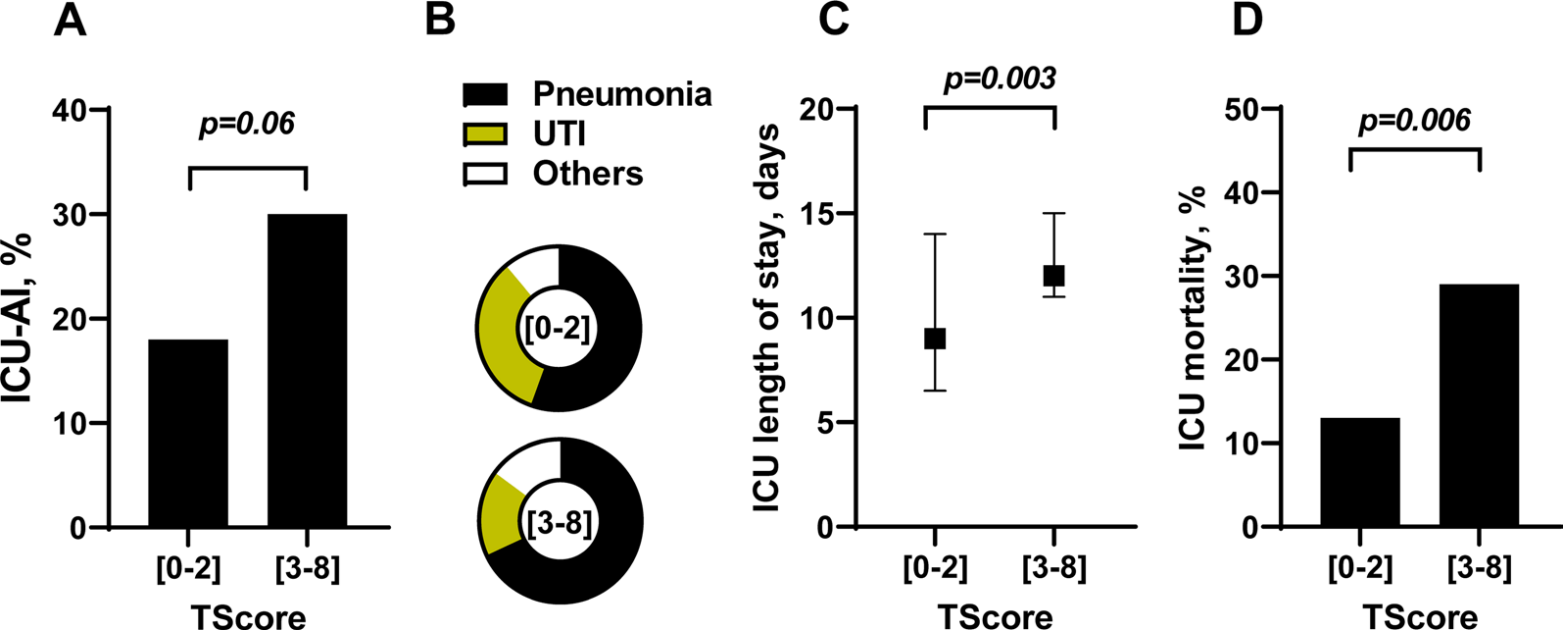
Clinical outcomes of high- and low-risk patients in the validation cohort using the TScore.** A** Intensive care unit acquired infection (ICU-AI) proportion in patients with a TScore between 0 and 2 (low risk) or upper or equal to 3 (high risk), **B** Distribution of the type of ICU-AI in patients with a TScore between 0 and 2 (low risk) or upper or equal to 3 (high risk) (UTI: urinary tract infections), **C** Median and interquartile duration of intensive care unit length of stay in patients with a TScore between 0 and 2 (low risk) or upper or equal to 3 (high risk), **D** intensive care unit mortality rate in patients with a TScore between 0 and 2 (low risk) or upper or equal to 3 (high risk)

ICU-acquired infections were more frequent in high-risk patients (30% vs. 18%, *p* = 0.06) (Fig. 5B), with a lower proportion of urinary tract infections (Fig. 5C). The median ICU length of stay was longer in high-risk patients (12 days, 25th–75th IQR: 8–20 vs. 9 days, 25th–75th IQR: 7–14, *p* = 0.003) (Fig. 5D). ICU mortality was also higher in high-risk patients (20% vs. 13%, *p* = 0.006) (Fig. 5E).

## Discussion

Two groups of patients with different risks of poor outcome, including ICU-acquired infections, mortality and length of ICU stay, were identified using a transcriptomic score based on the expression level of 8 genes. Relative to low-risk patients, patients in the high-risk group had an increased risk of ICU-acquired infections, even after adjustment for SOFA score, longer duration of ICU stay and higher mortality. High-risk assignment at day 5–7 was associated with lower mHLA-DR values and higher circulating IL10 values.

Owing to early identification of sepsis, rapid initiation of antibiotics and organ support therapies, many septic patients now survive the early phase of sepsis. However, they are therefore exposed to a higher risk of secondary infections that are associated with several markers of immune dysfunction such as downregulated monocytic HLA-DR expression [30]. Despite a robust pathophysiological rationale, several randomized clinical trials have failed in showing a beneficial effect of immune modulating therapies over the last decades [31]. One of the hypothesis for this failure relies on the important heterogeneity in septic patients and suggests to stratify patients on the basis of immune status in order to identify those who are more likely to benefit from these therapies [32]. Such stratification requires reliable, relevant, rapid and decision-making monitoring tools.

With this aim, assessment of the global differential gene expression within blood has provided encouraging results in the early phase of sepsis [27, 33, 34]. However, whether most of the studies fostered on mortality [23, 27, 33, 35], only a few genes were reported as relevant biomarkers to predict ICU-acquired infections [25]. Indeed, it is difficult to identify genes associated with a higher risk of ICU-acquired infection and several hypotheses can be formulated to explain it. First, blood cellular composition and gene expression vary rapidly over time depending on patient-related and management-related parameters. Second, the onset of sepsis remains unknown in most cases. Therefore, the simultaneous assessment of the expression of several genes appears as an interesting approach in order to pick up a signal whatever the time when the sample is performed [10]. To do so, we took advantages of the advent of multiplex molecular platforms such as the bioMérieux FilmArray System that allows the realization of rapid and reliable evaluation of the expressions of several genes. We performed our analysis between 5 and 7 after ICU admission, which accurately captures the impact of both the underlying condition and critical care interventions on the patient's immune status. This time frame also allows to shift away from the initially dysregulated immunity and reach the moment where some patients exhibit clear sign of return to homeostasis while others keep being dysregulated [36]. Furthermore, transcriptomic results were unaffected by the occurrence of ICU-acquired infections that were absent at this timepoint.

Over the last years, machine learning (ML) algorithms have gained increasing interest for classification, unsupervised clustering or dimensionality reduction tasks of large data sets. ML tools use data-driven algorithms and statistical models to analyze data sets and then, draw inferences from identified patterns or make predictions based on them. However, ML algorithms based on gene-expression performances in predicting ICU-acquired infections remain similar to usual severity scores [37]. These poor results could be related to the mismatch between two main assumptions of the ML algorithms and the characteristics of the clinical and biological data from which the algorithms are derived. First, ML algorithms assume the absence of covariate shift between training and test set. This assumption is frequently violated in ICU given the important heterogeneity in patient’s characteristics. Second, ML models intrinsically generate different features importance among dependent variables in order to fit as well as possible to the data. This assumption may be responsible for data overfitting and therefore, limit generalization of the models.

Based on these observations, we built the TScore, considering that 1/ each gene is independently associated with ICU-acquired infection and has an equal importance in the score without any weighting and 2/ that genes included in the score are mutually independent. This possibly allows for the capture of different pathophysiological mechanisms that would reflect an alteration of the immune system. This simplistic approach does not take into account the possible interactions between genes in signaling pathways that may be involved. However, given the differences among studies regarding the setting and the heterogeneity in septic patients, we believe this approach is appropriate in capturing relevant information in heterogeneous patients.

We found that the TScore was associated with the occurrence of ICU-acquired infections independently of the clinical variables usually associated with it. Furthermore, we found in high-risk patients biological features of immune suppression, with low levels of monocytic HLA-DR, high rate of immature neutrophils and high levels of the anti-inflammatory cytokine IL-10. These data suggest that the TScore is able to identify patients at risk of ICU-acquired infections based on circulating immune alterations. Therefore, our results suggest that this TScore could be used as a stratifying tool to identify patients likely to benefit from immune therapies. This approach could also allow to propose specific strategies for the prevention of ICU-AI, for instance by specifically monitoring microbiological carriage or by implementing dedicated prophylactic measures.

This score was build using a cohort of critically ill patients admitted in the ICU with various etiologies, including severe infections, burns, trauma and surgery. In line with previously published results, we postulated that immune alterations in circulating cells are shared among various medical conditions [11]. We then were able to validate this approach on a cohort of patients exhibiting exclusively a severe infection, which shows the robustness of the score.

This study acknowledges several limitations. First, we provided validation in an historical independent cohort and not in a prospectively collected cohort. Second, even if the results were repeated on a validation cohort, our results need to be evaluated in a large cohort and performed in a prospective way to confirm the performances of this test. Third, our results are based on whole-blood leukocyte populations that may differ among patients. Therefore, we cannot exclude that our results rather reflect differences in leukocytes populations rather than intracellular gene-expression differences. Fourth, the PCR multiplex was performed at day 5–7 after ICU admission. This limits the diagnostic performance of this test to the time period to which it is performed. The TScore must be validated and/or adapted at several time points, which should be possible thanks to the agility and plasticity of the platform.

## Conclusions

Using a multiplex molecular platform, we built a transcriptomic score based on simultaneous assessment of the expression of eight genes at day 5–7 after ICU admission that identifies a subgroup of patients at high risk to develop ICU-acquired infections. The host response that is detected in high-risk patients is associated with known biomarkers of immune dysfunction and provide a useful and reliable companion diagnostic tool to further develop immune modulating drugs evaluation in sepsis.

## Supplementary Information

**Additional file 1: Table S1.** Table summarizing diagnostic performances of identified genes in the discovery cohort, based on OOP ROC threshold computation. **Table S2.** Table comparing low and high-risk patients, for previously reported biomarkers as being associated with poor outcomes in ICU. **Fig. S1.** ROC (Receiver Operating Characteristics) curve with AUC (area under the curve) value of the ability of the OOP-Tscore obtained at day 5–7 to distinguish between patients that will develop or not at least an ICU-acquired infection during their ICU stay

## Data Availability

The original contributions presented in the study are included in the article. Further inquiries can be directed to the corresponding author.

## Abbreviations

AUC: Area under the curve
COVID-19: Coronavirus disease 2019
ICU-AI: Intensive care unit acquired infections
ML: Machine learning
PCR: Polymerase chain reaction
ROC: Receiver operator characteristics
SOFA: Sequential organ failure assessment
VAP: Ventilator-associated pneumonia
