# Supplementary material for: Identification of a sub-group of critically ill patients with high risk of intensive care unit-acquired infections and poor clinical course using a transcriptomic score
Source: Crit Care. 2023 Apr 21;27:158. doi: 10.1186/s13054-023-04436-3 (PMC10119529; doi:10.1186/s13054-023-04436-3)

**Supplementary Table 1:** Table summarizing diagnostic performances of identified genes in the discovery cohort, based on OOP ROC threshold computation.

| **Genes** | **AUC** | **Cut-off value OOP** | **Specificity** | **Sensibility** |
| --- | --- | --- | --- | --- |
| **C3AR1** | 0.73 (0.65 - 0.80) | > 0.3608 | 63.57 (54.98 - 71.37) | 82.98 (69.86 - 91.11) |
| **CD177** | 0.76 (0.68 – 0.84) | > 2.867 | 72.87 (64.62 - 79.80) | 68.09 (53.83 - 79.60) |
| **CX3CR1** | 0.73 (0.64 – 0.81) | < -1.833 | 65.89 (57.36 - 73.51) | 68.09 (53.83 - 79.60) |
| **IFNγ** | 0.73 (0.64 – 0.81) | < -5.300 | 65.12 (56.56 - 72.80) | 65.96 (51.67 - 77.83) |
| **IL1R2** | 0.77 (0.70 – 0.84) | > 3.894 | 69.77 (61.37 - 77.03) | 85.11 (72.31 - 92.59) |
| **S100A9** | 0.71 (0.62 – 0.79) | > -0.1892 | 70.54 (62.17 - 77.72) | 65.96 (51.67 - 77.83) |
| **TDRD9** | 0.74 (0.65 – 0.82) | > 1.117 | 68.22 (59.75 - 75.63) | 61.70 (47.43 - 74.21) |
| **ZAP70** | 0.74 (0.65 – 0.81) | < 2.483 | 65.12 (56.56 - 72.80) | 76.60 (62.78 - 86.40) |

**Supplementary Table 2:** Table comparing low and high risk patients, for previously reported biomarkers as being associated with poor outcomes in ICU.

|  | **Low-risk patients (n=89)** | **High-risk patients (n=87)** | **p.value** |
| --- | --- | --- | --- |
| **mHLA-DR, AB/C** | 10446 (7311 - 14878) | 4873 (3416 - 7121) | **<0.001** |
| **IL-10, pg/mL** | 7.37 (5.86 - 10.60) | 14.24 (11.36 - 23.03) | **<0.001** |
| **Immature neutrophils, %** | 7.92 (4.16 - 17.85) | 34.73 (22.27 - 68.31) | **<0.001** |

**Supplementary Figure 1:** ROC (Receiver Operating Characteristics) curve with AUC (area under the curve) value of the ability of the OOP-Tscore obtained at day 5-7 to distinguish between patients that will develop or not at least an ICU-acquired infection during their ICU stay.


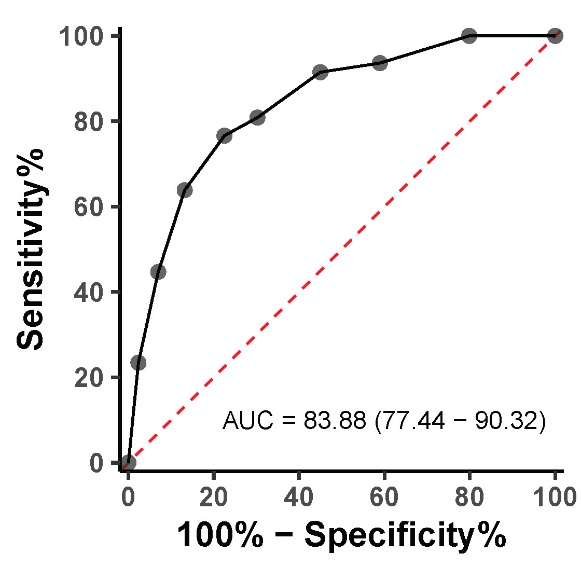

Supplement: Supplementary file 1 — Additional file 1: Table S1. Table summarizing diagnostic performances of identified genes in the discovery cohort, based on OOP ROC threshold computation. Table S2. Table comparing low and high-risk patients, for previously reported biomarkers as being associated with poor outcomes in ICU. Fig. S1. ROC (Receiver Operating Characteristics) curve with AUC (area under the curve) value of the ability of the OOP-Tscore obtained at day 5–7 to distinguish between patients that will develop or not at least an ICU-acquired infection during their ICU stay [file 13054_2023_4436_MOESM1_ESM.docx]
